# RNA m6A methylation regulatory mechanism of resveratrol in premature senescence cells

**DOI:** 10.1002/fsn3.4487

**Published:** 2024-09-30

**Authors:** Xinyu Zhang, Chenyu Zhu, Luyun Zhang, Luyi Tan, Wenli Cheng, Min Li, Xingtan Zhang, Wenjuan Zhang, Wenji Zhang

**Affiliations:** ^1^ Department of Public Health and Preventive Medicine, School of Medicine Jinan University Guangzhou Guangdong People's Republic of China; ^2^ Key Laboratory of Crop Genetic Improvement of Guangdong Province Crops Research Institute, Guangdong Academy of Agricultural Sciences Guangzhou Guangdong People's Republic of China; ^3^ National Key Laboratory for Tropical Crop Breeding, Shenzhen Branch, Guangdong Laboratory for Lingnan Modern Agriculture, Genome Analysis Laboratory of the Ministry of Agriculture Agricultural Genomics Institute at Shenzhen, Chinese Academy of Agricultural Sciences Shenzhen Guangdong People's Republic of China

**Keywords:** human embryonic lung fibroblasts, hydrogen peroxide, premature senescence, resveratrol, RNA m6A methylation

## Abstract

Resveratrol, a natural compound found in various plants, is known for its anti‐inflammatory, antioxidant, and senescence‐delaying properties. RNA N6‐methyladenosine (m6A) methylation plays a crucial role in oxidative stress and premature cellular senescence processes and is closely associated with age‐related disorders. However, the anti‐premature senescence via RNA m6A methylation mechanism of resveratrol is still not fully understood. In this study, based on premature senescence model of human embryonic lung fibroblasts (HEFs) induced by hydrogen peroxide (H_2_O_2_), a widely accepted model of premature senescence caused by oxidative stress, we explored the anti‐aging regulatory effects of resveratrol at the RNA m6A methylation level. Our data suggested that resveratrol significantly delayed premature senescence by increasing cell viability, reducing SA‐β‐gal blue staining rate, ROS levels, and senescence‐associated secretory phenotypes (SASP) expression in HEFs. Meanwhile, resveratrol increased the whole RNA methyltransferases activity and the overall m6A level during senescence. Furthermore, three genes *CCND2*, *E2F1*, and *GADD45B* have been identified as the main ones regulating premature by resveratrol. Specifically, it decreased *E2F1*, *GADD45B* RNA m6A methylation level, and increased *CCND2* level in premature cells. Our study provided new clues for exploring the mechanism and application of resveratrol in the field of premature aging.

## Graphical abstract

The 10 μmol/L resveratrol intervention promoted the proliferation of HEFs, restored the viability of prematurely senescent cells, reduced the intracellular reactive oxygen species and senescence‐associated secretory phenotype, and alleviated the premature senescence phenotype. Resveratrol could modulate the expression levels of m6A RNA methylation‐related enzymes and proteins in H_2_O_2_‐induced premature cellular senescence and alleviate premature senescence‐related RNA methyltransferase activity with reduced overall m6A levels. Three key senescence genes, CCND2, E2F1, and GADD45B, were identified. Resveratrol upregulated CCND2, and downregulated E2F1 and GADD45B m6A methylation levels.

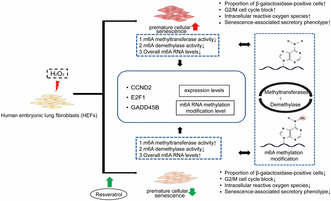

## 1 INTRODUCTION

Resveratrol (C_14_H_12_O_3_), a natural phenolic compound, has three polar hydroxyl groups and planar structure (Vernousfaderani et al., 2021). As an active small molecule widely used in the stilbene family, resveratrol is present in grapes, peanuts, plums, and berries (Breuss et al., 2019). And it has a wide range of biological effects, including senility‐delaying, immune‐boosting, diabetes‐relieving, and so on (Nadile et al., 2022; Ren et al., 2021). The mechanisms of the anti‐aging effect for resveratrol include oxidative stress, inflammation inhibition, and regulating mitochondrial function and apoptosis (Pyo et al., 2020). For example, in photoaging HaCat cells and aging mice model, resveratrol alleviated the photoaging induced by ultraviolet radiation B irradiation by regulating MAPK, COX2, and Nrf2 signaling pathways (Cui et al., 2022). Resveratrol could delay the oocyte degeneration of aging mice induced by oxidative stress, increase the expression of anti‐aging molecule Sirtuin1, decrease the level of intracellular ROS, and improve the function of mitochondria (Liang et al., 2018). These activities are attributed to the structural characteristics of resveratrol. Its free hydroxyl group could protect cells from peroxidation and enhance the activity of endogenous antioxidant enzymes, with the property of ROS scavenging (Repossi et al., 2020). And the high affinity of the hydrophobic domain was able to interact with many key proteins in the signal networks, such as PPARγ, NQO2, and COX1 (Britton et al., 2015). In addition, it also changed the acetylation pattern of proteins and affected the histone methylation level (Lv et al., 2018; Pinheiro et al., 2019). Methylation of histone H3 at the Lys9 site was related to transcriptional inhibition (Saccani & Natoli, 2002).

Epigenetics could connect exogenous environmental factors with the genetic background of the body and played an important regulatory role in the occurrence and development of cell senescence (Kennedy et al., 2014). RNA m6A methylation was a dynamic process, regulated by methyltransferase, demethylase, and binding protein (Cao et al., 2016). The involvement of m6A in the regulation of cellular senescence such as HEFs, human umbilical vein endothelial cells, human mesenchymal stem cells, and β cells had been demonstrated (Gao et al., 2023; Wu et al., 2022). The combination of dasatinib and quercetin played a role in human umbilical vein endothelial cells senescence induced by lipopolysaccharide, through regulating RNA m6A (Fan et al., 2022). The m6A methyltransferase (MELLT3/MELLT14 and WTAP) had been demonstrated to control the abundance of aging‐related proteins p21 and p27 by modifying their mRNA through methylation (Casella et al., 2019). Plant compounds like resveratrol had been shown to regulate m6A methylation. Dietary supplementation with resveratrol in mice could alter m6A methylation levels and attenuate high‐fat diet‐induced disturbances in hepatic lipid homeostasis (Wu et al., 2020).

Cell senescence was described as a state of limited ability of cell division when cultured in vitro, accompanied by a series of phenotypic changes such as cell morphology, gene expression, and function (Minamino et al., 2004). Replicative and premature senescence induced by stress (H_2_O_2_, ultraviolet, and hyperoxia) were the main types of cell senescence (Marrella et al., 2022). Senescent cells usually had increased cell size, slower metabolism, and permanent growth arrest, accompanied by increased secretion of various SASP such as chemokines, inflammatory factors, and growth factors (Childs et al., 2015; Ogrodnik, 2021; Rhinn et al., 2019). As the most representative substance in ROS, H_2_O_2_ was the signal molecule of normal physiological metabolism due to its good cell membrane permeability and high water solubility (Lismont et al., 2019). However, moderate exposure to H_2_O_2_ could induce premature senescence under certain conditions (Kim et al., 2022; Lee et al., 2022; Seo et al., 2019). HEFs were isolated from human embryonic lung tissue. H_2_O_2_‐induced HEFs was a common premature senescence model induced by oxidative stress, which had been used to explore the mechanism of cell senescence, evaluate aging‐related disease models, and develop anti‐aging intervention strategies (Wang, Gao, et al., 2021; Wu et al., 2022; Zhang et al., 2014).

It is necessary to investigate further how resveratrol influences m6A methylation modification during aging. Therefore, based on the H_2_O_2_‐induced premature senescence model, our study aimed to verify whether resveratrol could reverse the premature senescence state and the mechanism.

## 2 MATERIALS AND METHODS

### 2.1 Cell culture

HEFs were purchased from the Cell Resource Center of Institute of Basic Medicine, Chinese Academy of Medical Sciences. HEFs were cultured in DMEM medium supplemented with 10% fetal bovine serum (FBS) and 1% penicillin–streptomycin double antibiotics in a cell incubator (5% CO_2_, 37°C).

### 2.2 H_2_O_2_ treatment and resveratrol intervention

With H_2_O_2_ and resveratrol as intervention, the cells were divided into five groups according to different treatment methods, including a control group (22 population doubling levels cells, 22PDL), premature senescence initiation group (PSi), premature senescence persistence group (PSp), premature senescence initiation with resveratrol intervention group (PSi‐R), and premature senescence persistence with resveratrol intervention group (PSp‐R).

The H_2_O_2_‐induced premature senescence cell model was established, as previously described (Wu et al., 2022). The 22PDL cells were treated with 400 μmol/L H_2_O_2_ for 2 h per day. After continuous treatment for 4 days, the PSi group was obtained, and then normal culture for 7 days was used as the PSp group. PSi and PSp groups were pretreated with 10 μmol/L resveratrol for 24 h before the start of H_2_O_2_ exposure. The culture medium containing 10 μmol/L resveratrol was changed on Days 0, 2, 4, and 7 after H_2_O_2_ exposure for 2 h on the first day (daily resveratrol intervention for 22 h).

### 2.3 Cell viability test

HEFs in the logarithmic growth phase were diluted into 8 × 10^3^ cells/mL with culture medium. The 100 μL cell suspension was absorbed from each well and inoculated on a 96‐well plate. Resveratrol was formulated into 0, 5, 10, 20, 40, 50, 80, 100, and 200 μmol/L concentrations and added to corresponding wells. After incubated in the incubator for 24 h, 10 μL CCK8 reagent was added to each experimental well. The OD value was measured by an enzyme labeling instrument. The culture medium was used as the blank group and the cells treated without resveratrol was as the control group. The cell survival rate was calculated by the formula: cell viability (%) = [(OD_experimental group_−OD_blank group_)/(OD_control group_−OD_blank group_)] × 100%.

### 2.4 SA‐β‐gal staining

The cells were inoculated in a six‐well plate, and stained with SA‐β‐gal staining kit according to the scheme (Beyotime, Shanghai, China). The cells were washed with PBS and fixed with fixative solution for 15 min. The cells were then washed three times again with PBS and stained using staining working solution. Incubated cells in an incubator at 37°C for 12 h (ensure there was no CO_2_ in the environment). The staining condition was observed under an ordinary light microscope (Leica, Solms, Germany). The percentage of blue‐stained cells was calculated. Recorded the number of stain‐positive cells versus the total number of cells and calculated the percentage of blue‐stained cells.

### 2.5 Cell cycle assay

Propidium iodide DNA staining was used to analyze the cell cycle by flow cytometry. The cells were washed with PBS twice and digested them with trypsin. After fixation and centrifugation to obtain the cell pellet, propidium iodide staining solution was added for staining. Flow cytometry (Agilent, Santa Clara, CA, USA) was used to detect red fluorescence and light scattering at an excitation wavelength of 488 nm.

### 2.6 Intracellular ROS content

Intracellular ROS content was measured using the ROS Detection Assay Kit (Biovision, Milpitas, CA, USA). In a 12‐well plate, 3 × 10^5^ cells per hole were inoculated overnight. Except that only 500 μL serum was added to the control group, 500 μL 1 × ROS Label (i.e., DCFH‐DA probe) diluted with serum‐free medium was added to each well. A multifunction microplate reader (BIO⁃TEK, Winooski, VT, USA) was used to detect the average fluorescence intensity at Ex/Em = 495/529 nm. Then, the total protein was extracted, and the protein concentration was determined by the BCA method. ROS content = (average fluorescence intensity/ average protein mass)−(average fluorescence intensity of control hole/ average protein mass of control hole). According to the formula, the intracellular ROS content was calculated.

### 2.7 q‐PCR

Total RNA from the cells was extracted with Trizol (Invitrogen, Carlsbad, CA, USA). Subsequently, cDNA was synthesized by using the PrimeScipt Master Mix (TaKaRa, Kyodo, Japan). Using the National Center for Biotechnology Information (NCBI) and ORIGENE websites to find the desired gene sequences, primer synthesis was entrusted to Guangzhou Aiji Biotechnology Co., Ltd. The *GAPDH* gene was used as an internal reference gene. The primers used in the experiment included *IL‐6*, *IL‐8*, *VEGF*, *MMP1*, *METTL3*, *METTL4*, *METTL14*, *WTAP*, *KIAA1429*, *FTO*, *ALKBH5*, *YTHDC1*, *YTHDC2*, *YTHDF1*, *YTHDF2*, *hnRNPA2B1*, *hnRNPC*, *CCND2*, *E2F1*, and *GADD45B*. The primer sequences of each gene were shown in Table S1. SYBR Premix Ex Taq™ II Kit (Tli RNaseH Plus, TaKaRa, Kyodo, Japan) kit was used for PCR reaction. The q‐PCR was conducted with a fluorescence quantitative PCR instrument (CFX connect, BIORAD, Hercules, CA, USA). The relative mRNA expression was calculated by using the 2−^∆∆Ct^ method.

### 2.8 Protein extraction and western blot

Total protein from HEFs was lysed in RIPA lysis buffer (Beyotime, Shanghai, China). Protein concentrations were measured by using the bicinchoninic acid (BCA) method. Proteins from total lysates were separated on 10% SDS‐polyacrylamide gel electrophoresis (SDS‐PAGE, Beyotime, Shanghai, China) and then transferred to polyvinylidene fluoride membrane (Millipore Corporation, Burlington, MA, USA), blocked at room temperature for 2 h. The antibody VEGF (sc‐7269), IL‐6 (sc‐28343), IL‐8 (sc‐8427), MMP1 (sc‐21731), E2F1 (sc‐251), CCND2 (sc‐53637), and GADD45B (sc‐377311) were purchased from Santa Cruz (CA, USA); METTL3 (ab195352), METTL4 (ab261735), METTL14 (ab98166), WTAP (ab195380), KIAA1429 (ab124892), FTO (ab124892), and hnRNPC (ab133607) from Abcam (Cambridge, UK); YTHDC1 (220159), YTHDC2 (220160), YTHDF1 (17479‐1‐AP), YTHDF2 (24744‐1‐AP17479‐1‐AP), YTHDF3 (25537‐1‐AP17479‐1‐AP), and hnRNPA2B1 (14813‐AP17479‐1‐AP) from Proteintech (Rosemont, IL, USA). The membranes were incubated with primary antibodies against at 1:1000 dilution, overnight at 4°C. After washing three times in TBST, the membranes were incubated with respective secondary antibodies anti‐rabbit IgG (ab6721, Abcam, Cambridge, UK) or anti‐mouse IgG (ab6789, Abcam, Cambridge, UK) at a dilution of 1:5000 for 1 h. Eventually, the membranes were rewashed three times with TBST. The semi‐quantitative detection of the strip was carried out by using the Image J software, and the optical density of each strip was recorded. The relative content of protein = the gray value of the target strip/the gray value of β‐actin.

### 2.9 ELISA assay for total RNA methylase activity

Epigenase m6A methylase activity/inhibition assay kit (EpiGentek, Farmingdale, NY, USA) was used to detect the activity of total RNA methyltransferase and demethylase. In an assay with this kit, the unique m6A substrate was stably coated on the strip wells. Active m6A methylases bound to and methylate m6A contained in the substrate. The un‐demethylated m6A in the substrate could be recognized with a high‐affinity m6A antibody, and the immuno‐signal was enhanced with enhancer solution. The ratio or amount of un‐demethylated m6A, which was inversely proportional to enzyme activity, could then be colorimetrically quantified through an ELISA‐like reaction. The following formula was used to calculate enzyme activity. Methyltransferase activity (ng/h/mg) = (OD_sample well−_OD_blank well_) × 1000/slope of the standard curve × amount of nuclear protein added × incubation time. Demethylase activity (ng/h/mg) = [(OD_control−_OD_blank_)−(OD_sample−_OD_blank_)] × slope of 1000/standard curve × amount of nuclear protein added × incubation time.

### 2.10 Overall m6A RNA methylation level detection

The m6A RNA methylation quantification kit (EpiGentek, Farmingdale, NY, USA) was used to detect the overall RNA m6A methylation level. RNA binding solution bound total RNA to the well plate. The m6A was detected by using capture and detection antibodies. The amount of RNA m6A was proportional to the measured OD value in microplate reader. The following formula was used for quantification: m6A(ng) = (OD_Sample_−OD_NC_)/Slope, m6A% = m6A(ng)/Sample RNA addition × 100%.

### 2.11 RNA sequencing and bioinformatics analysis

Total RNA from the cells was extracted with Trizol (Invitrogen, Carlsbad, CA, USA). After preparing the RNA samples of the cells of each treatment group, RNA sequencing, and quality inspection were completed by Guangzhou Chideo Biology Co., Ltd. R package “Limma” (V 3.56.2) was used to analyze the sequencing results. The *p* < .05 and | log_2_FC | > 0.5 genes were defined as differentially expressed genes (DEGs). R package “ClusterProfiler” (V 4.6.2) was used to analyze the differentially expressed genes by Gene Ontology (GO) and Kyoto Encyclopedia of Genes and Genomes (KEGG) enrichment analysis. The *p*‐value cutoff and *q*‐value cutoff were set to 1. String (V 11.5) online website (cn.string‐db.org) was used to draw the protein interaction network map.

### 2.12 Methylated RNA immunoprecipitation (MeRIP) q‐PCR

Magna merip™ m6A kit (Millipore, MMAS, Burlington, MA, USA) was used to detect overall levels of RNA m6A methylation, strictly following the instructions of the kit. First, the extracted RNA was segmented, and then the magnetic beads for immunoprecipitation were prepared and immunoprecipitated. RNA fragments were eluted and purified with miRNeasy® mini kit (QIAGEN, Germantown, USA), found the corresponding DNA sequence of mRNA on the UCSC website, and designed related gene primers online through the primer design website (https://bioinfo.ut.ee/primer3‐0.4.0/). The primer sequences were shown in Table S1. Finally, the RNA fragment was operated by q‐PCR separately.

### 2.13 Statistical analysis

The experiments were performed in three or more replicates, and the results were expressed as mean ± standard deviation. Statistical analysis was performed by using SPSS 20.0 software, and graphpad prism 9 was used to plot the relevant statistical graphs. One‐way ANOVA was used for comparison between multiple groups, and Tukey's test was used for two‐way comparison between groups, with a two‐sided test at *α* = .05. The *p* < .05 was considered as a statistically significant difference.

## 3 RESULTS

### 3.1 Resveratrol treatment modified cell biological characteristics

**FIGURE 1:**
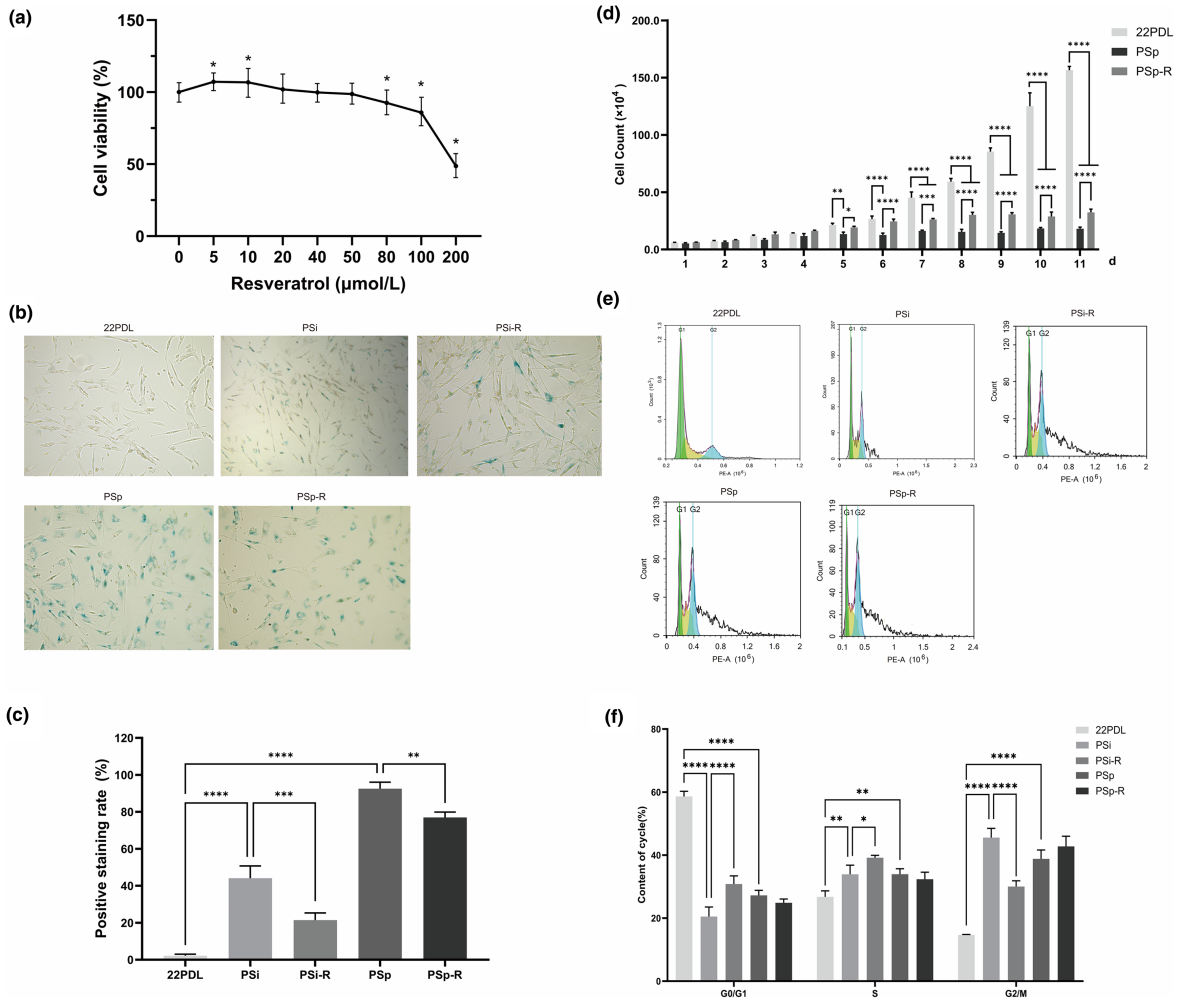
General biological characteristics of cells. (a) Effect of resveratrol on HEFs viability for 24 h. (b) Microscopic examination results of SA‐β‐gal staining. (c) Histogram of SA‐β‐gal positive staining rate in each group. (d) Cell counts over 11 days. (e) Cell cycle distribution. (f) Cell cycle histograms for each group. The data for each group are presented as the means ± SEMs (*n* = 3). *p < .05, **p < .01, ***p < .001, ****p < .0001.

First, we determined the effects of 0, 5, 10, 20, 40, 50, 80, 100, and 200 μmol/L resveratrol intervention in cells for 24 h on cell viability. The results showed that the concentrations of 5 and 10 μmol/L significantly increased cell viability, as shown in Figure 1a. In the subsequent experiments, 10 μmol/L resveratrol was chosen as the intervention concentration in order to reduce the experimental error.

SA‐β galactosidase staining showed a significant increase in blue‐stained cells in the PSi and PSp groups compared with that of 22PDL. And after resveratrol intervention, the rate of blue staining was reduced from Figure 1b,c.

Subsequently, we calculated the changes in cell number over 11 days. From Day 5 onward, the number of cells in the PSp group was lower compared with that of 22PDL group. And after resveratrol intervention, the cells in PSp‐R group increased compared with that of PSp group in Figure 1d.

The cell cycle results revealed a decrease in the proportion of G0/G1 phase and an increase in the proportion of cells in S and G2/M phases in the PSi and PSp groups compared with that of 22PDL group. After resveratrol intervention, the proportion of G0/G1 phase increased and the proportion of G2/M phase decreased in the PSi‐R group compared with that of PSi group in Figure 1e,f.

Therefore, these results indicated that resveratrol improved the HEFs viability, promoted cell proliferation, and regulated the senescent cell cycle.

### 3.2 Resveratrol reduced the level of ROS and SASP

**FIGURE 2:**
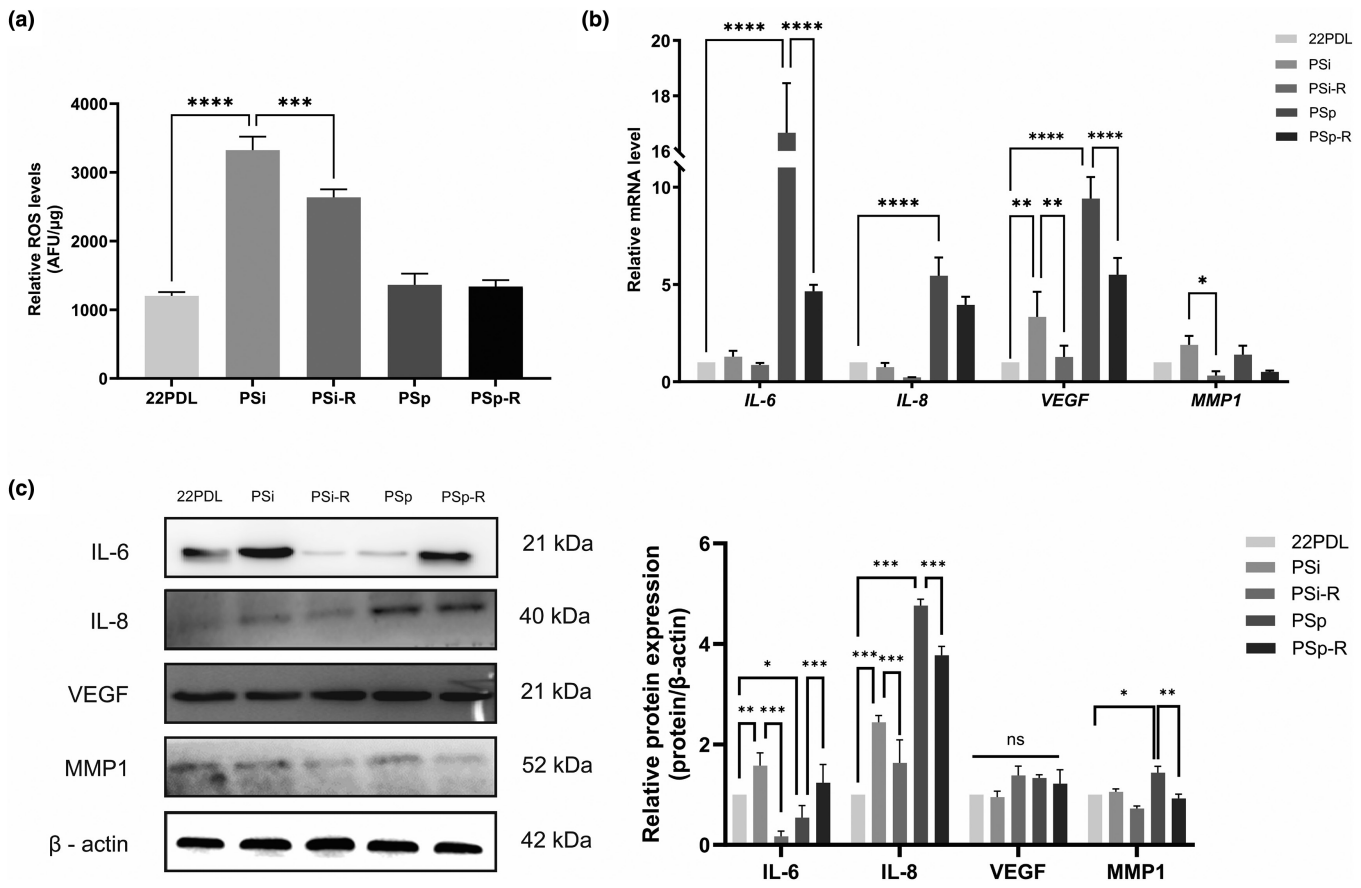
ROS level and SASP expression. (a) Relative quantification of total ROS in cells. (b) The mRNA expression level of SASP (*IL‐6*, *IL‐8*, *VEGF*, and *MMP1*). (c) The relative protein expression of SASP. The data for each group are presented as the means ± SEMs (*n* = 3). *p < .05, **p < .01, ***p < .001, ****p < .0001, ^ns^ *p* > .05.

The changes in intracellular ROS levels and SASP were assessed in this study. The results demonstrated that the intracellular ROS levels were elevated in the PSi group compared with that of 22PDL group, and decreased in the PSi‐R group after resveratrol treatment. And there was no significant difference in ROS levels between the PSp and PSp‐R groups as shown in Figure 2a.

Four indicators (*IL‐6*, *IL‐8*, *VEGF*, and *MMP1*) of SASP were detected. As for the mRNA level, following resveratrol intervention, the expressions of *VEGF* and *MMP1* in the PSi group as well as *IL‐6* and *VEGF* in the PSp group were significantly reduced as shown in Figure 2b.

Protein levels of IL‐8 were reduced in both intervention groups with statistically significant differences, and MMP1 decreased statistically in the PSp‐R group. However, there was no statistically significant difference between changes in VEGF before and after the intervention, as shown in Figure 2c.

### 3.3 Resveratrol affected methyltransferase activity and m6A level

**FIGURE 3:**
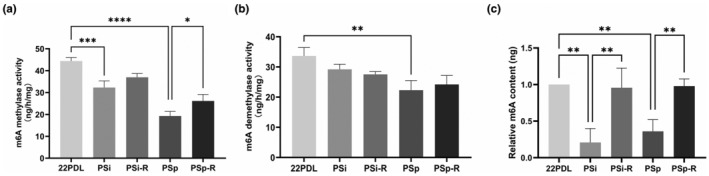
Changes of RNA m6A methylation microenvironment. (a) Activity of RNA m6A methyltransferases. (b) Activity of RNA m6A demethylases. (c) Overall RNA m6A methylation levels in cells. The data for each group are presented as the means ± SEMs (*n* = 3). *p < .05, **p < .01, ***p < .001, ****p < .0001.

The whole m6A methyltransferases activity, demethylases activity, and total m6A levels were assessed in each group. Methyltransferases activity was decreased in the two senescent groups compared with that of 22PDL group and was increased after resveratrol intervention as shown in Figure 3a. The decrease in demethylases activity was statistically significant in the PSp group and not in any of other groups in Figure 3b. As for total m6A levels, they decreased in both senescent groups and returned to almost normal following resveratrol intervention in Figure 3c.

### 3.4 The changes of mRNA and protein expression of the RNA m6A methylation regulatory factors

Furthermore, we conducted an analysis of the mRNA expression of key RNA methyltransferases (*METTL3*, *METTL4*, *METTL14*, *WTAP*, and *KIAA1429*), RNA demethylases (*FTO* and *ALKBH5*), and RNA binding proteins (*YTHDC1*, *YTHDC2*, *YTHDF1*, *YTHDF2*, *hnRNPA2B1*, and *hnRNPC*).

**FIGURE 4:**
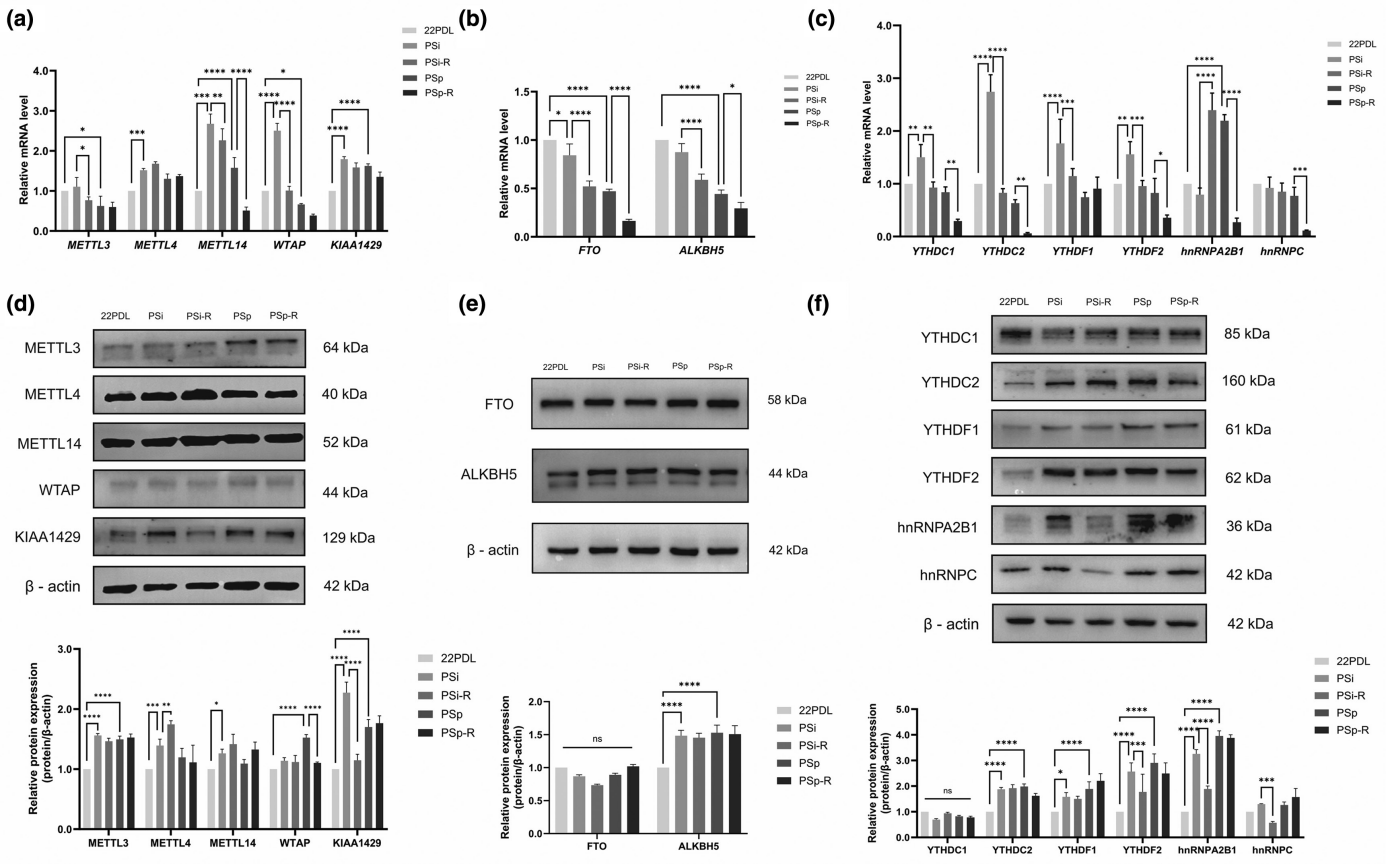
Changes in the RNA m6A methylation regulatory factors. (a) RNA m6A methyltransferase mRNA expression level. (b) RNA m6A demethylase mRNA expression level. (c) RNA m6A binding protein mRNA expression level. (d) RNA m6A methyltransferase relative protein expression. (e) RNA m6A demethylase relative protein expression. (f) RNA m6A binding protein relative protein expression. The data for each group are presented as the means ± SEMs (*n* = 3). *p < .05, **p < .01, ***p < .001, ****p < .0001, ^ns^ *p* > .05.

The *METTL14* mRNA was elevated in the two senescent groups and decreased after resveratrol intervention, and the difference was statistically significant. *WTAP* mRNA was elevated in the PSi group and decreased to the same level as that of the 22PDL group in the PSi‐R group, while the mRNA of *METTL3*, *METTL4*, and *KIAA1429* did not have statistically significant changes before and after intervention, as shown in Figure 4a. In the PSp group, the mRNAs of *FTO* and *ALKBH5* were both decreased compared with the mRNA levels of 22PDL group, and the decrease was more significant after the intervention, as shown in Figure 4b. *YTHDC1*, *YTHDC2*, *YTHDF1*, and *YTHDF2* were elevated in the PSi group, and *hnRNPA2B1* was elevated in the PSp group. After the corresponding resveratrol intervention, the decreases of all the above mRNAs were statistically different, as shown in Figure 4c.

Subsequently, we evaluated the protein expression of the regulatory system for RNA m6A methylation. Methyltransferases METTL4 and KIAA1429 were elevated in the PSi group compared with that of 22PDL group. After resveratrol intervention, METTL4 was elevated and KIAA1429 was decreased, as shown in Figure 4d. Demethylation enzymes FTO and ALKBH5 didn't change significantly before and after intervention, as shown in Figure 4e. The reading proteins YTHDF2 and hnRNPA2B1 were elevated in the PSi group and decreased after intervention, and the changes in other reading proteins were shown in Figure 4f.

It was evident that resveratrol reduced mRNA levels of METTL14, WTAP, FTO, YTHDC1, YTHDC2, YTHDF1, and YTHDF2 while also reducing protein levels of WTAP, KIAA1429, YTHDF2, hnRNPA2B1, and hnRNPC in senescent cells. Additionally, it increased the protein level of METTL4. The m6A methylation‐related enzymes and binding proteins exhibited variations in both mRNA and protein expression levels, indicating complex regulatory relationships at different stages of cellular senescence.

### 3.5 Identification and enrichment analysis of differentially expressed genes

**FIGURE 5:**
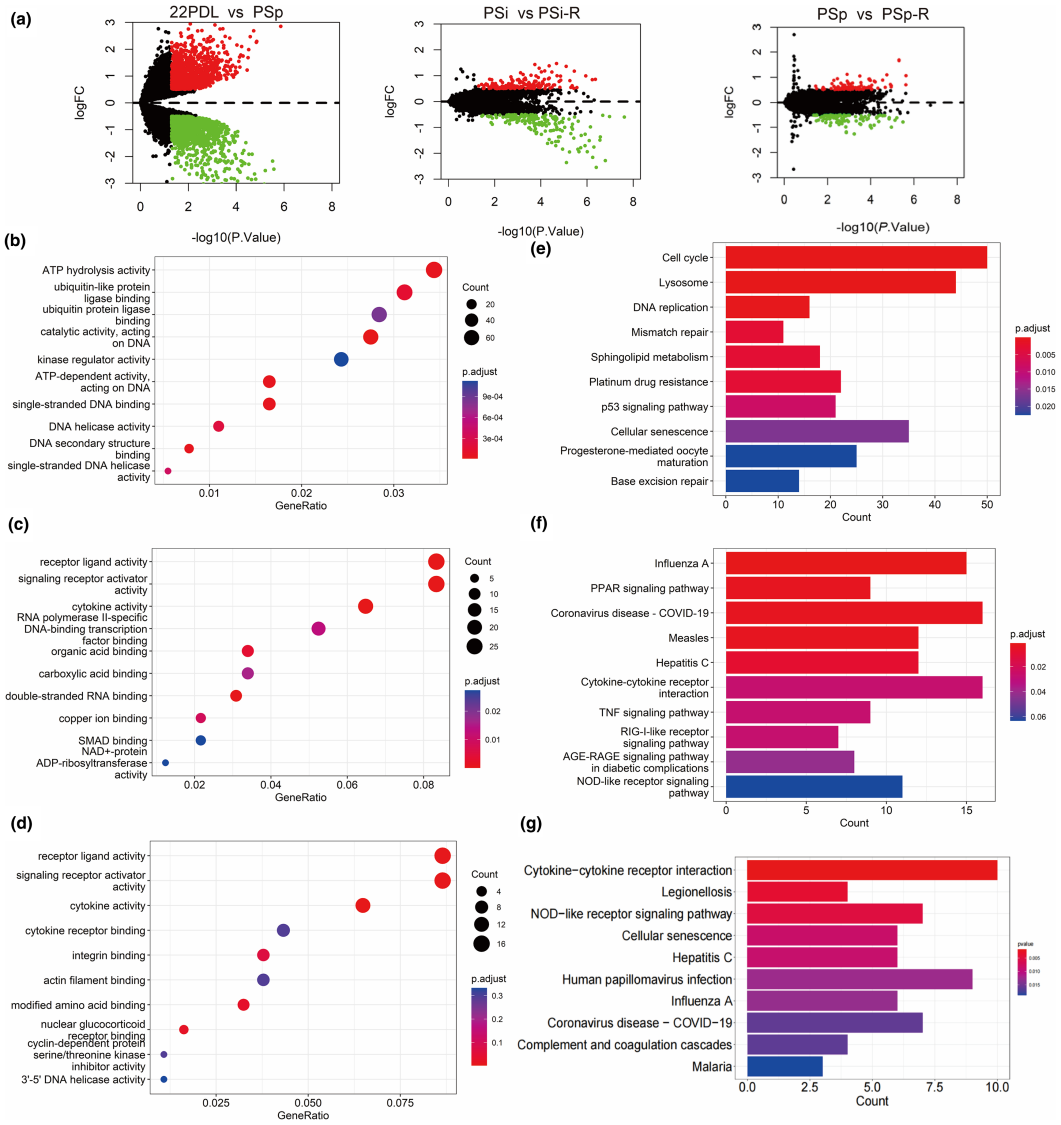
Identification and enrichment analysis of DEGs. (a) Volcanic map of different genes in different comparison groups. Red dots represent significantly upregulated genes, green dots represent significantly downregulated genes, and black dots represent genes with no statistically significant difference. (b) GO analyses of DEGs (22PDLvs PSp). (c) GO analyses of DEGs (PSi vs. PSi‐R). (d) GO analyses of DEGs (PSp vs. PSp‐R). (e) KEGG analyses of DEGs (22PDLvs PSp). (f) KEGG analyses of DEGs (PSi vs. PSi‐R). (g) KEGG analyses of DEGs (PSp vs. PSp‐R).

In order to unravel the molecular mechanisms associated with aging, a differential gene expression analysis was performed to identify the aging process and changes in gene expression following resveratrol intervention. We identified 1616, 344, and 197 differentially expressed genes (DEGs) from the comparison between premature aging groups (22PDL vs. PSp) and the resveratrol intervention‐related groups (PSi vs. PSi‐R, PSp vs. PSp‐R) in Figure 5a. This suggested significant alterations in gene expression during HEFs senescence in response to resveratrol intervention in senescent cells.

The results of GO functional enrichment revealed enrichment in pathways such as ATP hydrolytic activity and single‐stranded DNA binding (22PDL vs. PSp) in Figure 5b, as well as cytokine activity, receptor ligand activity, signal receptor activator activity, and other functions (PSi vs. PSi‐R and PSp vs. PSP‐R) in Figure 5c,d. Subsequently, KEGG pathway enrichment analysis was conducted to study the main enrichment pathways of DEGs, including cell senescence, the p53 signaling pathway, and the cell cycle (22PDL vs. PSp) in Figure 5e, the PPAR signaling pathway (PSi vs. PSi‐R) in Figure 5f, and cellular senescence (PSp vs. PSP‐R) in Figure 5g. These results indicated the involvement of cytokines and receptor ligands in the development of cell senescence.

### 3.6 Expression of senescence‐related specific genes and the RNA m6A methylation modification

**FIGURE 6:**
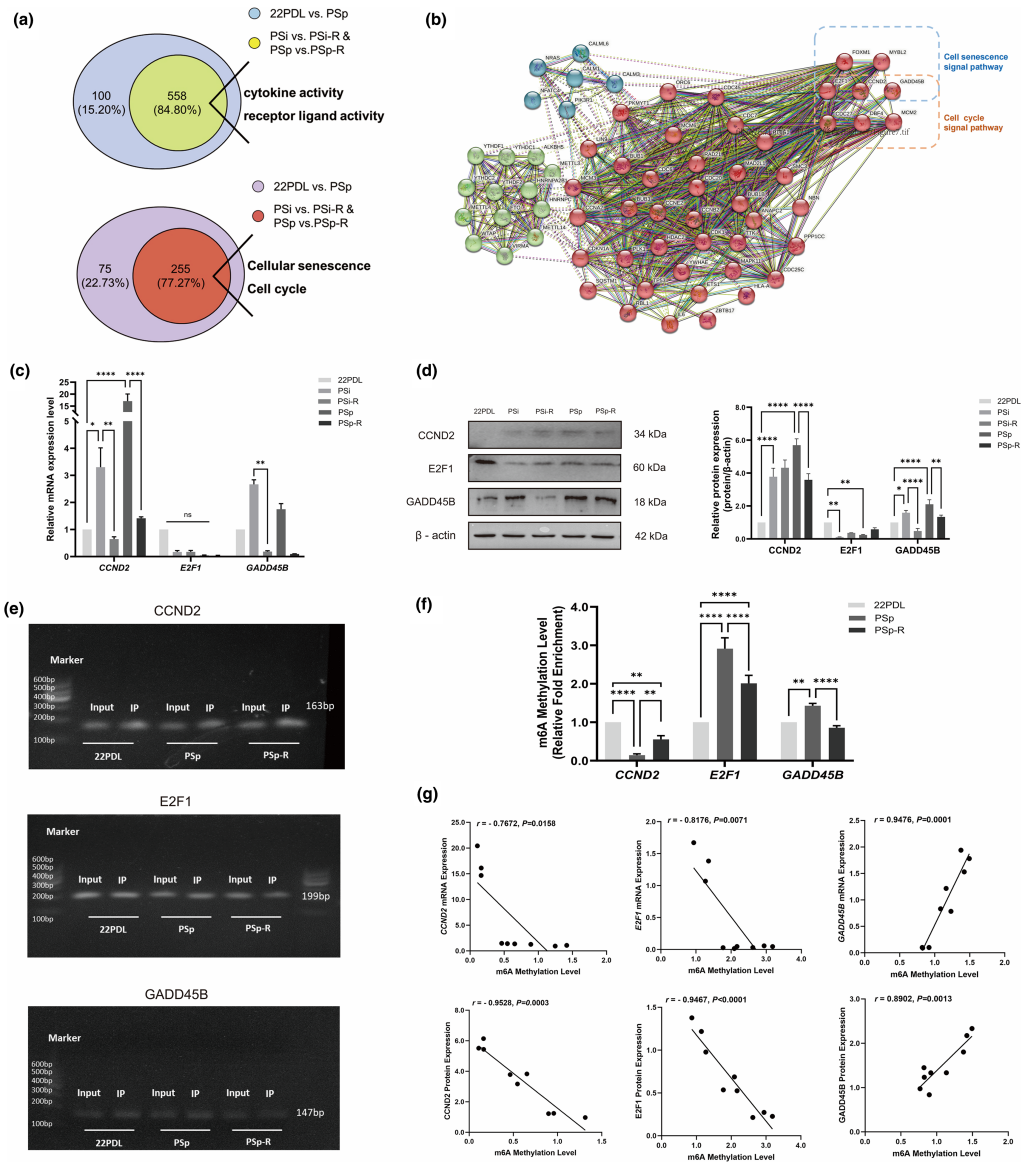
Screening of genes associated with aging. Expression of senescence‐related specific genes and the RNA m6A methylation modification. (a) Gene ontology enrichment of aging differential genes and intervention differential genes. Signaling pathways for enrichment of aging differential genes and intervention differential genes. (b) Network diagram of specific genes interacting with m6A regulator proteins (the solid line represented the direct relationship, and the dashed line represented the indirect relationship. Thicker wires indicated higher confidence. The green cluster was the m6A regulator, and the red and blue clusters were specific genes). (c) The mRNA expression levels of *CCND2*, *E2F1*, and *GADD45B*. (d) Protein expression levels of CCND2, E2F1, and GADD45B. (e) Gel electrophoresis of real‐time fluorescence quantitative PCR products of CCND2, E2F1, and GADD45B genes. (f) RNA m6A methylation levels of CCND2, E2F1, and GADD45B. (g) Correlation analysis of specific gene expression levels with their m6A methylation modifications. The data for each group are presented as the means ± SEMs (*n* = 3). *p < .05, **p < .01, ****p < .0001. ^ns^ *p* > .05.

The gene ontology enriched by resveratrol accounted for 84.80% of the results of aging differential gene set enrichment, while the proportion of enriched signaling pathways was 77.27% in Figure 6a. In this study, we focused on the intersection signaling pathways, hsa04218 cell senescence and hsa04110 cell cycle. The protein interaction network map of intersection genes and m6A regulatory factors revealed that m6A regulatory factors were indirectly associated with specific genes in Figure 6b. Given the observed regulatory phenotype of resveratrol on the cell cycle of aging cells in our preliminary tests, we focused on three genes—CCND2, E2F1, and GADD45B—which were involved in both the cell cycle and cellular senescence signaling pathways, and conducted an analysis and validation of them.

Regarding mRNA expression, the *CCND2* and *GADD45B* were lower in the PSi‐R and PSp‐R groups compared with that of the two aging groups, while the *E2F1* did not change significantly, as shown in Figure 6c. As for protein expression, CCND2 was elevated in the PSp group and lowered in the PSp‐R group after resveratrol intervention, and GADD45B was elevated in both aging groups and lowered after intervention in Figure 6d.

To understand the relationship between methylation and the regulation of mRNA and protein, we investigated the levels of m6A methylation in three genes during resveratrol intervention. The amplification maps of input samples and immunoprecipitation samples among groups showed that primers could successfully amplify PCR products in Figure 6e. In the PSp group, hypomethylation of *CCND2* and hypermethylation of *E2F1* and *GADD45B* were reversed after resveratrol intervention, as shown in Figure 6f.

The correlation between the level of RNA m6A methylation and the mRNA expression of specific genes in each treatment group showed that the mRNA expression level of CCND2 and E2F1 was negatively correlated with the level of m6A methylation, while the mRNA level of GADD45B was positively correlated with the level of m6A methylation. The correlation trend between CCND2, E2F1, and GADD45B proteins' expression and m6A methylation level was consistent with the above in Figure 6g.

Thus, the mRNA and protein changes of CCND2 and GADD45B were consistent and downregulated, but their methylation changes were opposite after resveratrol intervention. Therefore, the increased RNA m6A modification of CCND2 inhibited its post‐transcriptional translation, resulting in a decrease in protein level. The decrease of m6A modification level in GADD45B finally led to a decrease in protein level. The mRNA level of E2F1 did not change significantly, and the protein expression increased, indicating that the hypomethylation of E2F1 increased its protein expression.

## 4 DISCUSSION

**FIGURE 7:**
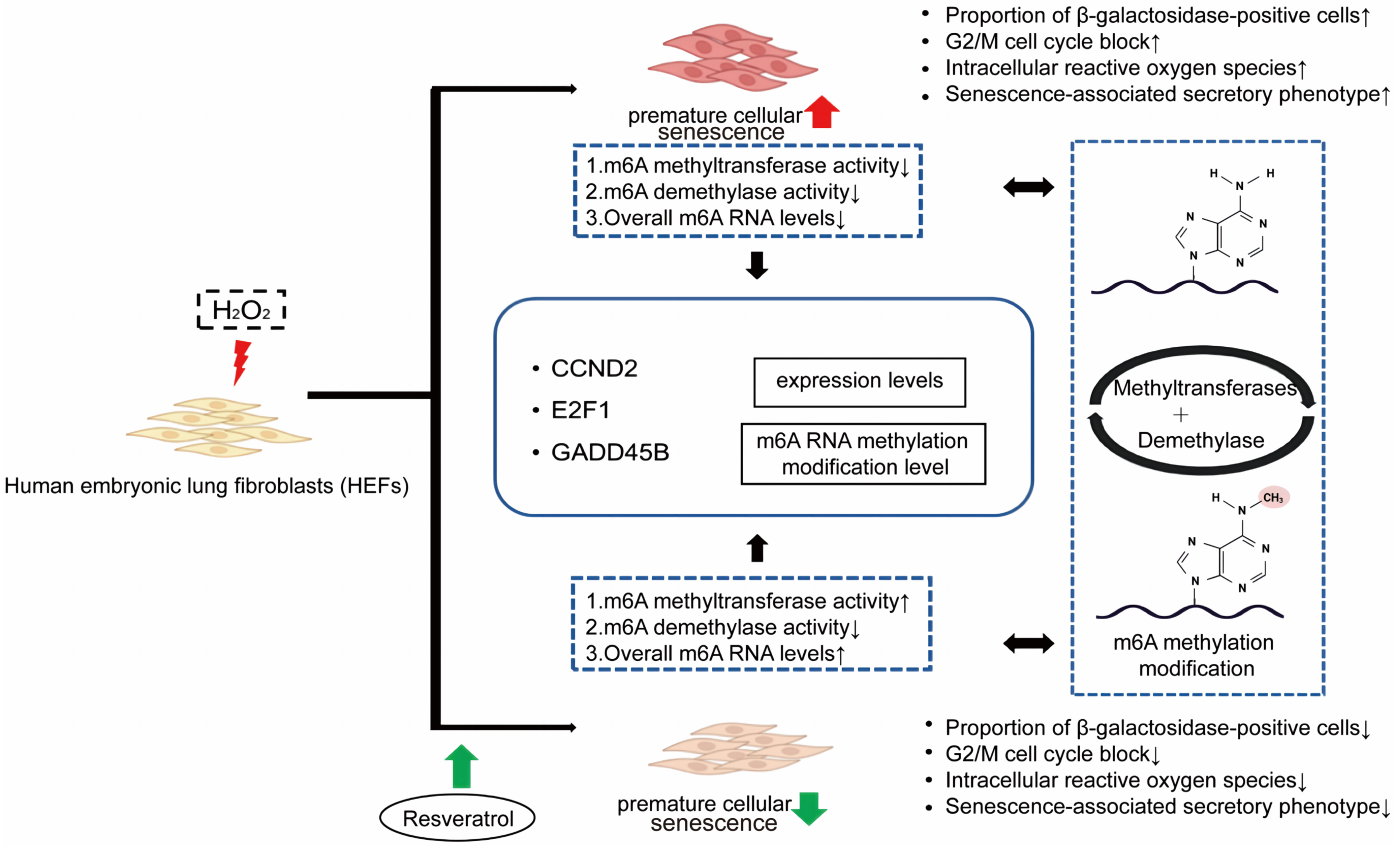
The mechanism of resveratrol on prematurely senescent HEFs.

Aging is an inevitable process, and with advancements in healthcare and declining fertility rates, population aging has become a global concern. Cellular senescence forms the scientific basis for the study of senescence. The hallmarks of cellular senescence include altered cell morphology, elevated SA‐β galactosidase activity, cell cycle arrest, redox imbalance, senescence‐associated secretory phenotypes, epigenetics, and other multifaceted alterations (Ogrodnik, 2021; Wei et al., 2024). Based on the original model of hydrogen peroxide‐induced premature senescence in HEFs, we separately determined the changes in their senescence markers and epigenetics in senescent cells under the effect of resveratrol intervention. The mechanism of resveratrol alleviation of premature aging by RNA m6A modification is shown in Figure 7. Specifically, the study comprehensively revealed the mechanism of resveratrol in delaying cellular premature senescence by enhancing general biological characteristics of cells, modulating RNA m6A‐related enzyme and protein levels, and altering the transcription and methylation levels of key aging genes.

Our study revealed that senescent HEFs undergo blockade at the G2/M phase. Similarly, G2/M phase block occurs in urothelial cells (Chien et al., 2000), retinal cells (Hwang et al., 2012; Kim et al., 2021), and melanoma cells (Tang et al., 2020) in response to H_2_O_2_ stimulation. Resveratrol regulated cell cycle disturbances by relieving G2/M phase arrest and increasing the proportion of cells entering the G0/G1 phase. This was similar to the effects of natural active substances such as anthocyanin oligomer (Hwang et al., 2012), pine tuber ethanol extract (Tang et al., 2020), and calendula extract (Alnuqaydan et al., 2015), which had been shown to alleviate cell cycle arrest during oxidative stress. The discovery of antioxidant plant compounds became crucial for maintaining redox homeostasis and slowing down the aging process. Resveratrol attenuated ROS release in a mouse model of pneumonia induced by pathogen infection (Chi et al., 2024). Cisplatin‐induced increased intracellular ROS in HEI‐OCI cells, and resveratrol reduced intracellular ROS levels, which was consistent with our findings (Wu et al., 2024). In senescent cells, resveratrol intervention significantly reduced ROS levels. This suggested that resveratrol could enhance the ability of HEFs to resist ROS imbalance induced by H_2_O_2_. Senescent cells secreted a variety of inflammatory cytokines, chemokines, growth factors, and proteases, and these molecules are referred to as senescence‐associated secretory phenotypes (Ortiz‐Montero et al., 2017). Our study found that resveratrol inhibited the production of intracellular SASP, including IL‐6, IL‐8, VEGF, and MMP1. Citrus flavanone naringenin, a polyphenol similar to resveratrol, also reduced the expression of SASP in senescent cells (Piragine et al., 2024). However, in the PSp group, IL‐6 protein expression was reduced, which might be due to the high secretion of IL‐6 into the cytosol.

Dynamic regulation of m6A modification levels played an important role in the occurrence and development of aging and aging‐related diseases. Resveratrol regulated the level of m6A modification during aging mainly by affecting methyltransferase activity. RNA m6A methylation levels were reduced in senescent HEF cells (Wu et al., 2022). Resveratrol had the effect of restoring overall methylation levels to normal. The RNA m6A methylation regulatory system had specific expression profile changes during H_2_O_2_‐induced premature aging and resveratrol intervention. During cellular senescence, m6A modifications regulated the level of proteins after the genes were transcribed, and this modification could enhance or reduce binding to specific regulatory factors (Li et al., 2023). Our study found that METTL14, WTAP, and KIAA1429 were downregulated by resveratrol and slowed down the aging process. WTAP could recruit METTL3 and METTL14 (Wan et al., 2022). WTAP was increased in senescent nucleus pulposus cells and promoted the interaction between NORAD and methyltransferase complexes (METTL3 and METTL14). Notably, the attenuation of the senescent state in TNF‐α‐treated nasopharyngeal carcinoma cells upon silencing WTAP underscores its therapeutic potential in mitigating age‐related or stress‐induced cellular decline (Li et al., 2022). In urothelial carcinoma, METTL4 could mediate hypoxia‐induced urothelial mesenchymal transformation and tumor metastasis (Hsu et al., 2022). DNA m6A methylation of METTL4 played an important role in mouse lipogenesis (Tooley et al., 2023). KIAA1429 could influence disease progression through m6A modification in cancer, reproductive system diseases, and cardiovascular system diseases (Zhang et al., 2022). Overexpression of KIAA1429 was a predictor of poor tumor prognosis, and low expression was a predictor of poor prognosis in non‐tumor diseases (Zhang et al., 2022). FTO and ALKBH5 are RNA demethylases, which make m6A methylation reversible (Jia et al., 2011; Zheng et al., 2013). However, no alterations in FTO with ALKBH5 were seen in the resveratrol intervention. The regulatory role played by resveratrol on demethylating enzymes remains to be explored. RNA methylation‐binding proteins recognize RNA with m6A methylation modifications. Proteins with YTH domains, such as YTHDC1 and YTHDC2 in the nucleus, and YTHDF1‐3 in the cytoplasm, were important members of RNA methylation‐binding proteins in eukaryotes (Zhang et al., 2010), which could coordinate with other RNA methylation‐binding proteins such as hnRNPA2B1 and hnRNPC to regulate RNA metabolism (Alarcón et al., 2015). YTHDF2 dysregulation was often associated with cancer, but it has also been found that the locus corresponding to the (TG)n microsatellite in the YTHDF2 gene could play a potential role in human longevity (Wang & Lu, 2021). The hnRNPA2B1 enhanced m6A‐dependent stability of oncogene mRNAs and promoted cancer progression (Hao et al., 2023). Resveratrol played a role in slowing down the aging process by reducing the expression of YTHDF2 and hnRNPA2B1.

Given the significant detection of changes in the cell cycle, we focused on common genes involved in both the cell cycle and cell senescence pathways. The three screened genes, CCND2, E2F1, and GADD45B, were found to play a significant role in regulating cell life processes. CCND2 is a member of the type D cyclin family and is involved in cell cycle regulation, cell differentiation, and malignant transformation. The majority of epigenetic studies on this gene have concentrated on DNA promoter methylation (Blanchet et al., 2011). CCND2 encodes proteins that facilitate the G1 to S phase transition, thereby regulating the progression of the cell cycle. Nevertheless, a single study indicated that reducing the expression of CCND2 has only a minimal impact on cell division (Singh et al., 2009). Oxidative stress‐induced cellular senescence signals activate the p53 signaling pathway and downregulate the cell cycle‐related gene E2F1, leading to cell cycle arrest (Matuoka & Chen, 2002). RNA m6A regulatory mechanisms also regulate the expression of E2F1. The RNA demethylation enzyme FTO inhibits the level of E2F1 m6A modification and thus promotes the expression of downstream genes, which contributes to the progression of non‐small cell lung cancer (Wang, Li, et al., 2021). GADD45B is a member of the GADD45 family, involved in the process of cellular injury and stress response (Wang et al., 2022). The expression level of GADD45B could be regulated by m6A modification and promotes musculogenesis through activation of the MAPK pathway (Deng et al., 2021). In vitro and in vivo experiments had demonstrated that m6A modification of the GADD45B gene had a role in the resistance to cisplatin in the treatment of germ cell tumors (Miranda‐Gonçalves et al., 2021). In conclusion, resveratrol may mitigate the progression by modulating the methylation levels of CCND2, E2F1, and GADD45B involved in aging process, which in turn affected transcription and translation. It has been found that inhibition of the methyltransferase METTL3 reduced m6A modification on Slc1a5 and ameliorated osteoblast senescence (Liu et al., 2024). Metformin enhanced METTL14 expression and promoted m6A methylation, which attenuated H_2_O_2_‐induced apoptosis in NIT‐1 cells (Zhou et al., 2024). Therefore, resveratrol intervention was expected to be a new strategy to delay organismal aging by altering the activity of key enzymes and regulating the level of m6A modification of key genes in cell aging. It is especially crucial to investigate and clarify the mechanism by which resveratrol regulates RNA m6A methylation in order to develop possible anti‐aging nutrients.

## AUTHOR CONTRIBUTIONS

**Xinyu Zhang:** Conceptualization (equal); data curation (equal); formal analysis (equal); methodology (equal); visualization (equal); writing – original draft (lead). **Chenyu Zhu:** Conceptualization (equal); formal analysis (equal); methodology (equal); visualization (equal); writing – original draft (supporting). **Luyun Zhang:** Conceptualization (equal); formal analysis (equal); methodology (equal); visualization (equal); writing – original draft (supporting). **Luyi Tan:** Data curation (supporting); formal analysis (supporting). **Wenli Cheng:** Data curation (supporting); formal analysis (supporting). **Min Li:** Data curation (equal); formal analysis (equal). **Xingtan Zhang:** Conceptualization (equal); methodology (equal). **Wenjuan Zhang:** Funding acquisition (supporting); project administration (equal); resources (equal); supervision (equal); writing – review and editing (equal). **Wenji Zhang:** Funding acquisition (lead); project administration (equal); resources (equal); supervision (equal); writing – review and editing (equal).

## FUNDING INFORMATION

This work was supported by the Guangdong Basic and Applied Basic Research Foundation (2023A1515010353, 2021A1515011220); Special fund for scientific innovation strategy‐construction of high‐level Academy of Agriculture Sciences (R2022PY‐QY004, R2018YJ‐YB3002); Science and Technology Planning Project of Guangdong Province (2023B1212060038).

## CONFLICT OF INTEREST STATEMENT

The authors declare no conflict of interest.

## Supporting information


**Data S1.** Supporting Information.

## Data Availability

The data that support the findings of this study are available from the corresponding author upon reasonable request.

## References

[fsn34487-bib-0001] Alarcón, C. R. , Goodarzi, H. , Lee, H. , Liu, X. , Tavazoie, S. , & Tavazoie, S. F. (2015). HNRNPA2B1 is a mediator of m(6)A‐dependent nuclear RNA processing events. Cell, 162, 1299–1308. 26321680 10.1016/j.cell.2015.08.011 PMC4673968

[fsn34487-bib-0002] Alnuqaydan, A. M. , Lenehan, C. E. , Hughes, R. R. , & Sanderson, B. J. (2015). Extracts from Calendula officinalis offer in vitro protection against H_2_O_2_ induced oxidative stress cell killing of human skin cells. Phytotherapy Research, 29, 120–124. 25266574 10.1002/ptr.5236

[fsn34487-bib-0003] Blanchet, E. , Annicotte, J.‐S. , Lagarrigue, S. , Aguilar, V. , Clapé, C. , Chavey, C. , Fritz, V. , Casas, F. , Apparailly, F. , Auwerx, J. , & Fajas, L. (2011). E2F transcription factor‐1 regulates oxidative metabolism. Nature Cell Biology, 13, 1146–1152. 21841792 10.1038/ncb2309 PMC3849758

[fsn34487-bib-0004] Breuss, J. M. , Atanasov, A. G. , & Uhrin, P. (2019). Resveratrol and its effects on the vascular system. International Journal of Molecular Sciences, 20, 1523. 30934670 10.3390/ijms20071523 PMC6479680

[fsn34487-bib-0005] Britton, R. G. , Kovoor, C. , & Brown, K. (2015). Direct molecular targets of resveratrol: Identifying key interactions to unlock complex mechanisms. Annals of the New York Academy of Sciences, 1348, 124–133. 26099829 10.1111/nyas.12796

[fsn34487-bib-0006] Cao, G. , Li, H.‐B. , Yin, Z. , & Flavell, R. A. (2016). Recent advances in dynamic m6A RNA modification. Open Biology, 6, 160003. 27249342 10.1098/rsob.160003 PMC4852458

[fsn34487-bib-0007] Casella, G. , Tsitsipatis, D. , Abdelmohsen, K. , & Gorospe, M. (2019). mRNA methylation in cell senescence. Wiley Interdisciplinary Reviews: RNA, 10, e1547. 31144457 10.1002/wrna.1547 PMC8474013

[fsn34487-bib-0008] Chi, F. , Cheng, C. , Zhang, M. , Su, B. , Hou, Y. , & Bai, G. (2024). Resveratrol targeting NRF2 disrupts the binding between KEAP1 and NRF2‐DLG motif to ameliorate oxidative stress damage in mice pulmonary infection. Journal of Ethnopharmacology, 332, 118353. 38762209 10.1016/j.jep.2024.118353

[fsn34487-bib-0009] Chien, M. , Rinker‐Schaeffer, C. , & Stadler, W. M. (2000). A G2/M growth arrest response to low‐dose intermittent H_2_O_2_ in normal uroepithelial cells. International Journal of Oncology, 17, 425–432. 10938379 10.3892/ijo.17.3.425

[fsn34487-bib-0010] Childs, B. G. , Durik, M. , Baker, D. J. , & van Deursen, J. M. (2015). Cellular senescence in aging and age‐related disease: From mechanisms to therapy. Nature Medicine, 21, 1424–1435. 10.1038/nm.4000 PMC4748967 26646499

[fsn34487-bib-0011] Cui, B. , Wang, Y. , Jin, J. , Yang, Z. , Guo, R. , Li, X. , Yang, L. , & Li, Z. (2022). Resveratrol treats UVB‐induced Photoaging by anti‐MMP expression, through anti‐inflammatory, antioxidant, and Antiapoptotic properties, and treats Photoaging by upregulating VEGF‐B expression. Oxidative Medicine and Cellular Longevity, 2022, 6037303. 35028009 10.1155/2022/6037303 PMC8752231

[fsn34487-bib-0012] Deng, K. , Fan, Y. , Liang, Y. , Cai, Y. , Zhang, G. , Deng, M. , Wang, Z. , Lu, J. , Shi, J. , Wang, F. , & Zhang, Y. (2021). FTO‐mediated demethylation of GADD45B promotes myogenesis through the activation of p38 MAPK pathway. Molecular Therapy—Nucleic Acids, 26, 34–48. 34513292 10.1016/j.omtn.2021.06.013 PMC8408560

[fsn34487-bib-0013] Fan, T. , Du, Y. , Zhang, M. , Zhu, A. R. , & Zhang, J. (2022). Senolytics cocktail Dasatinib and quercetin alleviate human umbilical vein endothelial cell senescence via the TRAF6‐MAPK‐NF‐κB Axis in a YTHDF_2_‐dependent manner. Gerontology, 68, 920–934. 35468611 10.1159/000522656

[fsn34487-bib-0014] Gao, P. , Yao, F. , Pang, J. , Yin, K. , & Zhu, X. (2023). M 6A methylation in cellular senescence of age‐associated diseases. Acta Biochimica et Biophysica Sinica Shanghai, 55, 1168–1183. 10.3724/abbs.2023107 PMC10449638 37394885

[fsn34487-bib-0015] Hao, W. , Chen, Z. , Tang, J. , Yang, R. , Gao, W.‐Q. , & Xu, H. (2023). hnRNPA2B1 promotes the occurrence and progression of hepatocellular carcinoma by downregulating PCK1 mRNA via a m6A RNA methylation manner. Journal of Translational Medicine, 21, 861. 38017546 10.1186/s12967-023-04704-4 PMC10683354

[fsn34487-bib-0016] Hsu, K.‐W. , Lai, J. C.‐Y. , Chang, J.‐S. , Peng, P.‐H. , Huang, C.‐H. , Lee, D.‐Y. , Tsai, Y. C. , Chung, C. J. , Chang, H. , Chang, C. H. , Chen, J. L. , Pang, S. T. , Hao, Z. , Cui, X. L. , He, C. , & Wu, K. J. (2022). METTL4‐mediated nuclear N6‐deoxyadenosine methylation promotes metastasis through activating multiple metastasis‐inducing targets. Genome Biology, 23, 249. 36461076 10.1186/s13059-022-02819-3 PMC9716733

[fsn34487-bib-0018] Hwang, J.‐W. , Kim, E.‐K. , Lee, S.‐J. , Kim, Y.‐S. , Moon, S.‐H. , Jeon, B.‐T. , Sung, S. H. , Kim, E. T. , & Park, P. J. (2012). Antioxidant activity and protective effect of anthocyanin oligomers on H₂O₂‐triggered G2/M arrest in retinal cells. Journal of Agricultural and Food Chemistry, 60, 4282–4288. 22380882 10.1021/jf205321j

[fsn34487-bib-0019] Jia, G. , Fu, Y. , Zhao, X. , Dai, Q. , Zheng, G. , Yang, Y. , Yi, C. , Lindahl, T. , Pan, T. , Yang, Y. G. , & He, C. (2011). N6‐methyladenosine in nuclear RNA is a major substrate of the obesity‐associated FTO. Nature Chemical Biology, 7, 885–887. 22002720 10.1038/nchembio.687 PMC3218240

[fsn34487-bib-0020] Kennedy, B. K. , Berger, S. L. , Brunet, A. , Campisi, J. , Cuervo, A. M. , Epel, E. S. , Franceschi, C. , Lithgow, G. J. , Morimoto, R. I. , Pessin, J. E. , Rando, T. A. , Richardson, A. , Schadt, E. E. , Wyss‐Coray, T. , & Sierra, F. (2014). Aging: A common driver of chronic diseases and a target for novel interventions. Cell, 159, 709–713. 25417146 10.1016/j.cell.2014.10.039 PMC4852871

[fsn34487-bib-0021] Kim, D. H. , Kim, J.‐H. , Hwangbo, H. , Kim, S. Y. , Ji, S. Y. , Kim, M. Y. , Cha, H. J. , Park, C. , Hong, S. H. , Kim, G. Y. , Park, S. K. , Jeong, J. W. , Kim, M. Y. , Choi, Y. H. , & Lee, H. (2021). Spermidine attenuates oxidative stress‐induced apoptosis via blocking Ca^2+^ overload in retinal pigment epithelial cells independently of ROS. International Journal of Molecular Sciences, 22, 1361. 33572992 10.3390/ijms22031361 PMC7866386

[fsn34487-bib-0022] Kim, H.‐J. , Kim, W.‐J. , Shin, H.‐R. , Yoon, H.‐I. , Moon, J.‐I. , Lee, E. , Lim, J. M. , Cho, Y. D. , Lee, M. H. , Kim, H. G. , & Ryoo, H. M. (2022). ROS‐induced PADI2 downregulation accelerates cellular senescence via the stimulation of SASP production and NFκB activation. Cellular and Molecular Life Sciences, 79, 155. 35218410 10.1007/s00018-022-04186-5 PMC8882118

[fsn34487-bib-0023] Lee, J.‐J. , Ng, S.‐C. , Hsu, J.‐Y. , Liu, H. , Chen, C.‐J. , Huang, C.‐Y. , & Kuo, W. W. (2022). Galangin reverses H_2_O_2_‐induced dermal fibroblast senescence via SIRT1‐PGC‐1α/Nrf2 signaling. International Journal of Molecular Sciences, 23, 1387. 35163314 10.3390/ijms23031387 PMC8836071

[fsn34487-bib-0024] Li, G. , Ma, L. , He, S. , Luo, R. , Wang, B. , Zhang, W. , Song, Y. , Liao, Z. , Ke, W. , Xiang, Q. , Feng, X. , Wu, X. , Zhang, Y. , Wang, K. , & Yang, C. (2022). WTAP‐mediated m6A modification of lncRNA NORAD promotes intervertebral disc degeneration. Nature Communications, 13, 1469. 10.1038/s41467-022-28990-6 PMC8933458 35304463

[fsn34487-bib-0025] Li, N. , Luo, R. , Zhang, W. , Wu, Y. , Hu, C. , Liu, M. , Jiang, D. , Jiang, Z. , Zhao, X. , Wang, Y. , & Li, Q. (2023). IL‐17A promotes endothelial cell senescence by up‐regulating the expression of FTO through activating JNK signal pathway. Biogerontology, 24, 99–110. 36463389 10.1007/s10522-022-09999-2

[fsn34487-bib-0026] Liang, Q.‐X. , Lin, Y.‐H. , Zhang, C.‐H. , Sun, H.‐M. , Zhou, L. , Schatten, H. , Sun, Q. Y. , & Qian, W. P. (2018). Resveratrol increases resistance of mouse oocytes to postovulatory aging in vivo. Aging (Albany NY), 10, 1586–1596. 30036861 10.18632/aging.101494 PMC6075442

[fsn34487-bib-0027] Lismont, C. , Koster, J. , Provost, S. , Baes, M. , Van Veldhoven, P. P. , Waterham, H. R. , & Fransen, M. (2019). Deciphering the potential involvement of PXMP2 and PEX11B in hydrogen peroxide permeation across the peroxisomal membrane reveals a role for PEX11B in protein sorting. Biochimica et Biophysica Acta, Biomembranes, 1861, 182991. 31129117 10.1016/j.bbamem.2019.05.013

[fsn34487-bib-0028] Liu, X.‐W. , Xu, H.‐W. , Yi, Y.‐Y. , Zhang, S.‐B. , Chang, S.‐J. , Pan, W. , & Wang, S. J. (2024). Inhibition of Mettl3 ameliorates osteoblastic senescence by mitigating m6A modifications on Slc1a5 via Igf2bp2‐dependent mechanisms. Biochimica et Biophysica Acta (BBA) ‐ Molecular Basis of Disease, 1870, 167273. 38844111 10.1016/j.bbadis.2024.167273

[fsn34487-bib-0029] Lv, Y.‐J. , Yang, Y. , Sui, B.‐D. , Hu, C.‐H. , Zhao, P. , Liao, L. , Chen, J. , Zhang, L. Q. , Yang, T. T. , Zhang, S. F. , & Jin, Y. (2018). Resveratrol counteracts bone loss via mitofilin‐mediated osteogenic improvement of mesenchymal stem cells in senescence‐accelerated mice. Theranostics, 8, 2387–2406. 29721087 10.7150/thno.23620 PMC5928897

[fsn34487-bib-0030] Marrella, V. , Facoetti, A. , & Cassani, B. (2022). Cellular senescence in immunity against infections. International Journal of Molecular Sciences, 23, 11845. 36233146 10.3390/ijms231911845 PMC9570409

[fsn34487-bib-0031] Matuoka, K. , & Chen, K. Y. (2002). Transcriptional regulation of cellular ageing by the CCAAT box‐binding factor CBF/NF‐Y. Ageing Research Reviews, 1, 639–651. 12362892 10.1016/s1568-1637(02)00026-0

[fsn34487-bib-0032] Minamino, T. , Miyauchi, H. , Yoshida, T. , Tateno, K. , Kunieda, T. , & Komuro, I. (2004). Vascular cell senescence and vascular aging. Journal of Molecular and Cellular Cardiology, 36, 175–183. 14871544 10.1016/j.yjmcc.2003.11.010

[fsn34487-bib-0033] Miranda‐Gonçalves, V. , Lobo, J. , Guimarães‐Teixeira, C. , Barros‐Silva, D. , Guimarães, R. , Cantante, M. , Braga, I. , Maurício, J. , Oing, C. , Honecker, F. , Nettersheim, D. , Looijenga, L. H. J. , Henrique, R. , & Jerónimo, C. (2021). The component of the m6A writer complex VIRMA is implicated in aggressive tumor phenotype, DNA damage response and cisplatin resistance in germ cell tumors. Journal of Experimental & Clinical Cancer Research, 40, 268. 34446080 10.1186/s13046-021-02072-9 PMC8390281

[fsn34487-bib-0034] Nadile, M. , Retsidou, M. I. , Gioti, K. , Beloukas, A. , & Tsiani, E. (2022). Resveratrol against cervical cancer: Evidence from in vitro and in vivo studies. Nutrients, 14, 5273. 36558430 10.3390/nu14245273 PMC9787601

[fsn34487-bib-0035] Ogrodnik, M. (2021). Cellular aging beyond cellular senescence: Markers of senescence prior to cell cycle arrest in vitro and in vivo. Aging Cell, 20, e13338. 33711211 10.1111/acel.13338 PMC8045927

[fsn34487-bib-0036] Ortiz‐Montero, P. , Londoño‐Vallejo, A. , & Vernot, J.‐P. (2017). Senescence‐associated IL‐6 and IL‐8 cytokines induce a self‐ and cross‐reinforced senescence/inflammatory milieu strengthening tumorigenic capabilities in the MCF‐7 breast cancer cell line. Cell Communication and Signaling, 15, 17. 28472950 10.1186/s12964-017-0172-3 PMC5418812

[fsn34487-bib-0037] Pinheiro, D. M. L. , de Oliveira, A. H. S. , Coutinho, L. G. , Fontes, F. L. , de Medeiros Oliveira, R. K. , Oliveira, T. T. , Faustino, A. L. F. , Lira da Silva, V. , de Melo Campos, J. T. A. , Lajus, T. B. P. , de Souza, S. J. , & Agnez‐Lima, L. F. (2019). Resveratrol decreases the expression of genes involved in inflammation through transcriptional regulation. Free Radical Biology & Medicine, 130, 8–22. 30366059 10.1016/j.freeradbiomed.2018.10.432

[fsn34487-bib-0038] Piragine, E. , De Felice, M. , Germelli, L. , Brinkmann, V. , Flori, L. , Martini, C. , Calderone, V. , Ventura, N. , Da Pozzo, E. , & Testai, L. (2024). The *citrus* flavanone naringenin prolongs the lifespan in *C. elegans* and slows signs of brain aging in mice. Experimental Gerontology, 194, 112495. 38897393 10.1016/j.exger.2024.112495

[fsn34487-bib-0039] Pyo, I. S. , Yun, S. , Yoon, Y. E. , Choi, J.‐W. , & Lee, S.‐J. (2020). Mechanisms of aging and the preventive effects of resveratrol on age‐related diseases. Molecules, 25, 4649. 33053864 10.3390/molecules25204649 PMC7587336

[fsn34487-bib-0040] Ren, B. , Kwah, M. X.‐Y. , Liu, C. , Ma, Z. , Shanmugam, M. K. , Ding, L. , Xiang, X. , Ho, P. C. L. , Wang, L. , Ong, P. S. , & Goh, B. C. (2021). Resveratrol for cancer therapy: Challenges and future perspectives. Cancer Letters, 515, 63–72. 34052324 10.1016/j.canlet.2021.05.001

[fsn34487-bib-0041] Repossi, G. , Das, U. N. , & Eynard, A. R. (2020). Molecular basis of the beneficial actions of resveratrol. Archives of Medical Research, 51, 105–114. 32111491 10.1016/j.arcmed.2020.01.010

[fsn34487-bib-0042] Rhinn, M. , Ritschka, B. , & Keyes, W. M. (2019). Cellular senescence in development, regeneration and disease. Development, 146, dev151837. 31575608 10.1242/dev.151837

[fsn34487-bib-0043] Saccani, S. , & Natoli, G. (2002). Dynamic changes in histone H3 Lys 9 methylation occurring at tightly regulated inducible inflammatory genes. Genes & Development, 16, 2219–2224. 12208844 10.1101/gad.232502 PMC186673

[fsn34487-bib-0044] Seo, E. , Kang, H. , Choi, H. , Choi, W. , & Jun, H. (2019). Reactive oxygen species‐induced changes in glucose and lipid metabolism contribute to the accumulation of cholesterol in the liver during aging. Aging Cell, 18, e12895. 30609251 10.1111/acel.12895 PMC6413652

[fsn34487-bib-0045] Singh, S. , Dhaliwal, N. , Crawford, R. , & Xiao, Y. (2009). Cellular senescence and longevity of osteophyte‐derived mesenchymal stem cells compared to patient‐matched bone marrow stromal cells. Journal of Cellular Biochemistry, 108, 839–850. 19693768 10.1002/jcb.22312

[fsn34487-bib-0046] Tang, D. , Yan, R. , Sun, Y. , Kai, G. , Chen, K. , & Li, J. (2020). Material basis, effect, and mechanism of ethanol extract of Pinellia ternata tubers on oxidative stress‐induced cell senescence. Phytomedicine, 77, 153275. 32659678 10.1016/j.phymed.2020.153275

[fsn34487-bib-0047] Tooley, J. G. , Catlin, J. P. , & Tooley, C. E. S. (2023). METTLing in stem cell and cancer biology. Stem Cell Reviews and Reports, 19, 76–91. 36094754 10.1007/s12015-022-10444-7 PMC10563446

[fsn34487-bib-0048] Vernousfaderani, E. K. , Akhtari, N. , Rezaei, S. , Rezaee, Y. , Shiranirad, S. , Mashhadi, M. , Hashemi, A. , Khankandi, H. P. , & Behzad, S. (2021). Resveratrol and colorectal cancer: A molecular approach to clinical researches. Current Topics in Medicinal Chemistry, 21, 2634–2646. 34749615 10.2174/1568026621666211105093658

[fsn34487-bib-0049] Wan, L. , Liu, J. , Huang, C. , Zhu, Z. , Li, F. , Sun, G. , Wang, K. , Li, S. , Ma, X. , Chen, X. , & Yuan, W. (2022). Role of m6A modification and novel circ_0066715/miR‐486‐5p/ ETS1 axis in rheumatoid arthritis macrophage polarization progression. Aging (Albany NY), 14, 10009–10026. 36541909 10.18632/aging.204439 PMC9831719

[fsn34487-bib-0050] Wang, D. , Li, Y. , Xu, X. , Zhao, S. , Wang, Z. , Yang, J. , Zhang, X. , Pan, J. , Wang, Y. , & Liu, M. (2022). FTO knockdown alleviates hypoxia‐induced PC12 cell injury by stabilizing GADD45B in an IGF2BP2‐dependent manner. Biochemical and Biophysical Research Communications, 619, 166–172. 35803057 10.1016/j.bbrc.2022.06.039

[fsn34487-bib-0051] Wang, J. , & Lu, A. (2021). The biological function of m6A reader YTHDF2 and its role in human disease. Cancer Cell International, 21, 109. 33593354 10.1186/s12935-021-01807-0 PMC7885220

[fsn34487-bib-0052] Wang, Y. , Gao, J. , Wu, F. , Lai, C. , Li, Y. , Zhang, G. , Peng, X. , Yu, S. , Yang, J. , Wang, W. , Zhang, W. , & Yang, X. (2021). Biological and epigenetic alterations of mitochondria involved in cellular replicative and hydrogen peroxide‐induced premature senescence of human embryonic lung fibroblasts. Ecotoxicology and Environmental Safety, 216, 112204. 33845364 10.1016/j.ecoenv.2021.112204

[fsn34487-bib-0053] Wang, Y. , Li, M. , Zhang, L. , Chen, Y. , & Zhang, S. (2021). m6A demethylase FTO induces NELL2 expression by inhibiting E2F1 m6A modification leading to metastasis of non‐small cell lung cancer. Molecular Therapy ‐ Oncolytics, 21, 367–376. 34169146 10.1016/j.omto.2021.04.011 PMC8190133

[fsn34487-bib-0054] Wei, H. , Xu, Y. , Lin, L. , Li, Y. , & Zhu, X. (2024). A review on the role of RNA methylation in aging‐related diseases. International Journal of Biological Macromolecules, 254, 127769. 38287578 10.1016/j.ijbiomac.2023.127769

[fsn34487-bib-0055] Wu, F. , Zhang, L. , Lai, C. , Peng, X. , Yu, S. , Zhou, C. , Zhang, B. , & Zhang, W. (2022). Dynamic alteration profile and new role of RNA m6A methylation in replicative and H_2_O_2_‐induced premature senescence of human embryonic lung fibroblasts. International Journal of Molecular Sciences, 23, 9271. 36012545 10.3390/ijms23169271 PMC9408987

[fsn34487-bib-0056] Wu, J. , Li, Y. , Yu, J. , Gan, Z. , Wei, W. , Wang, C. , Zhang, L. , Wang, T. , & Zhong, X. (2020). Resveratrol attenuates high‐fat diet induced hepatic lipid homeostasis disorder and decreases m6A RNA methylation. Frontiers in Pharmacology, 11, 568006. 33519432 10.3389/fphar.2020.568006 PMC7845411

[fsn34487-bib-0057] Wu, W. , Li, Y. , He, J. , Yang, J. , & Liu, Y. (2024). Resveratrol shields against cisplatin‐induced ototoxicity through epigenetic lncRNA GAS5 modulation of miR‐455‐5p/PTEN pathway. International Immunopharmacology, 138, 112464. 38917526 10.1016/j.intimp.2024.112464

[fsn34487-bib-0058] Zhang, W. , Hu, D. , Ji, W. , Yang, L. , Yang, J. , Yuan, J. , Xuan, A. , Zou, F. , & Zhuang, Z. (2014). Histone modifications contribute to cellular replicative and hydrogen peroxide‐induced premature senescence in human embryonic lung fibroblasts. Free Radical Research, 48, 550–559. 24528089 10.3109/10715762.2014.893580

[fsn34487-bib-0059] Zhang, X. , Li, M. , Xia, L. , & Zhang, H. (2022). The biological function of m6A methyltransferase KIAA1429 and its role in human disease. PeerJ, 10, e14334. 36389416 10.7717/peerj.14334 PMC9657180

[fsn34487-bib-0060] Zhang, Z. , Theler, D. , Kaminska, K. H. , Hiller, M. , de la Grange, P. , Pudimat, R. , Rafalska, I. , Heinrich, B. , Bujnicki, J. M. , Allain, F. H. T. , & Stamm, S. (2010). The YTH domain is a novel RNA binding domain. The Journal of Biological Chemistry, 285, 14701–14710. 20167602 10.1074/jbc.M110.104711 PMC2863249

[fsn34487-bib-0061] Zheng, G. , Dahl, J. A. , Niu, Y. , Fedorcsak, P. , Huang, C.‐M. , Li, C. J. , Vågbø, C. B. , Shi, Y. , Wang, W. L. , Song, S. H. , Lu, Z. , Bosmans, R. P. G. , Dai, Q. , Hao, Y. J. , Yang, X. , Zhao, W. M. , Tong, W. M. , Wang, X. J. , Bogdan, F. , … He, C. (2013). ALKBH5 is a mammalian RNA demethylase that impacts RNA metabolism and mouse fertility. Molecular Cell, 49, 18–29. 23177736 10.1016/j.molcel.2012.10.015 PMC3646334

[fsn34487-bib-0062] Zhou, S. , Yao, X. , Cheng, Y. , Xing, Y. , Sun, Y. , Hua, Q. , Wan, S. J. , & Meng, X. J. (2024). Metformin enhances METTL14‐mediated m6A methylation to alleviate NIT‐1 cells apoptosis induced by hydrogen peroxide. Heliyon, 10, e24432. 38312705 10.1016/j.heliyon.2024.e24432 PMC10835167

