# Supplementary material for: RNA m6A methylation regulatory mechanism of resveratrol in premature senescence cells
Source: Food Sci Nutr. 2024 Sep 30;12(11):9238–51. doi: 10.1002/fsn3.4487 (PMC11606896; doi:10.1002/fsn3.4487)
Supplement: Supplementary file 1 — Data S1. Supporting Information. [file FSN3-12-9238-s001.docx]

**Supplementary Information**

**RNA m6A methylation regulatory mechanism of resveratrol in premature senescence cells**

Xinyu Zhang^1, #^, Chenyu Zhu^1, #^, Luyun Zhang^1,^ ^#^, Luyi Tan^1^, Wenli Cheng^1,2^, Min Li^1^, Xingtan Zhang^3^, Wenjuan Zhang^1,^ ^*^, Wenji Zhang^2, *^

^1^ Department of Public Health and Preventive Medicine, School of Medicine, Jinan University, Guangzhou, Guangdong, 510632, P.R. China.

^2^ Key Laboratory of Crop Genetic Improvement of Guangdong Province, Crops Research Institute, Guangdong Academy of Agricultural Sciences, Guangzhou, Guangdong, 510640, P. R. China

^3^ National Key Laboratory for Tropical Crop Breeding, Shenzhen Branch, Guangdong Laboratory for Lingnan Modern Agriculture, Genome Analysis Laboratory of the Ministry of Agriculture, Agricultural Genomics Institute at Shenzhen, Chinese Academy of Agricultural Sciences, Shenzhen, Guangdong, 518120, P.R. China

^*^Correspondence should be addressed to:

Dr. Wenji Zhang, Key Laboratory of Crop Genetic Improvement of Guangdong Province, Crops Research Institute, Guangdong Academy of Agricultural Sciences, Guangzhou, Guangdong 510640, P. R. China, E-mail: zhangwenji@gdaas.cn

Dr. Wenjuan Zhang, Department of Public Health and Preventive Medicine, School of Medicine, Jinan University, Guangzhou, Guangdong 510632, P.R. China, E-mail: zwj2080@126.com

^#^ These authors contributed equally to this work.

| **Supplementary Table 1.** **Gene specific primer sequences** | | |
| --- | --- | --- |
| Primer Name | Forward primer Sequence (5'-3') | Reverse primer Sequence (5'-3') |
| **q-PCR primers** |  |  |
| *GAPDH* | GTCTCCTCTGACTTCAACAGCG | ACCACCCTGTTGCTGTAGCCAA |
| *IL-6* | GACAGCCACTCACCTCTTCA | AGTGCCTCTTTGCTGCTTTC |
| *IL-8* | CTCTCTTGGCAGCCTTCCTGA | CAATAATTTCTGTGTTGGCGC |
| *VEGF* | TGCTGTACCTCCACCATGCCA | CTGCAAGTACGTTCGTTTAAC |
| *MMP1* | CTGAAGGTGATGAAGCAGCC | AGTCCAAGAGAATGGCCGAG |
| *METTL3* | CTATCTCCTGGCACTCGCAAGA | GCTTGAACCGTGCAACCACATC |
| *METTL4* | ACAGGGTCTGCTTCCTGATG | GGAGGACAGTATTTTGTTGCCA |
| *METTL14* | CTGAAAGTGCCGACAGCATTGG | CTCTCCTTCATCCAGATACTTACG |
| *WTAP* | GCAACAACAGCAGGAGTCTGCA | CTGCTGGACTTGCTTGAGGTAC |
| *KIAA1429* | TGACCTTGCCTCACCAACTGCA | AGCAACCTGGTGGTTTGGCTAG |
| *FTO* | CCAGAACCTGAGGAGAGAATGG | CGATGTCTGTGAGGTCAAACGG |
| *ALKBH5* | CCAGCTATGCTTCAGATCGCCT | GGTTCTCTTCCTTGTCCATCTCC |
| *YTHDC1* | TCAGGAGTTCGCCGAGATGTGT | AGGATGGTGTGGAGGTTGTTCC |
| *YTHDC2* | GAAAGCTCCTGAACCTCCACCA | GGTTCTACTGGCAAGTCAGCCA |
| *YTHDF1* | CAAGCACACAACCTCCATCTTCG | GTAAGAAACTGGTTCGCCCTCAT |
| *YTHDF2* | TAGCCAGCTACAAGCACACCAC | CAACCGTTGCTGCAGTCTGTGT |
| *hnRNPA2B1* | CAGCAACCTTCTAACTACGGTCC | CACTGCCTCCTGGACCATAGTT |
| *hnRNPC* | TGGGCTGCTCTGTTCATAAGGG | CTCGGTTCACTTTTGGCTCTGC |
| *CCND2* | ATTGAACCATTTGGGATGGA | AGGAGCCAATCACCTGTGTC |
| *E2F1* | CTGCATGCACATACACACCA | GGTTTCCAGAGATGCTCACC |
| *GADD45B* | CCCTCGACAAGACCACACTT | TATGCTTCCCATCTCGCTCT |
| **MeRIP-q-PCR primers** |  |  |
| *CCND2* | ATTGAACCATTTGGGATGGA | AGGAGCCAATCACCTGTGTC |
| *E2F1* | CTGCATGCACATACACACCA | GGTTTCCAGAGATGCTCACC |
| *GADD45B* | CCCTCGACAAGACCACACTT | TATGCTTCCCATCTCGCTCT |
